# Atraumatic Polycompartment Syndrome Secondary to Cardiogenic Shock: A Case Report

**DOI:** 10.7759/cureus.44519

**Published:** 2023-09-01

**Authors:** Victor B Yang, Henry Shu, Manuj M Shah, Xiyu Zhao, Siam T Muquit, Marc Greenberg, Glenn Whitman, Sung-Min Cho, Bo Soo Kim, Babar Shafiq

**Affiliations:** 1 Critical Care, Johns Hopkins University School of Medicine, Baltimore, USA; 2 Orthopaedic Surgery, Johns Hopkins University School of Medicine, Baltimore, USA; 3 General Surgery, Johns Hopkins University School of Medicine, Baltimore, USA; 4 Internal Medicine, Johns Hopkins University School of Medicine, Baltimore, USA; 5 Cardiology, Johns Hopkins University School of Medicine, Baltimore, USA; 6 Cardiac Surgery, Johns Hopkins University School of Medicine, Baltimore, USA; 7 Neurology, Johns Hopkins University School of Medicine, Baltimore, USA

**Keywords:** compartment syndrome, intraabdominal hypertension, abdominal compartment syndrome, cardiogenic shock, polycompartment syndrome

## Abstract

We report the case of a 53-year-old male who developed polycompartment syndrome (PCS) secondary to cardiogenic shock. After suffering a cardiac arrest, a self-perpetuating cycle of intra-abdominal hypertension (IAH) and vital organ damage led to abdominal compartment syndrome (AbCS), which then contributed to the precipitation of extremity compartment syndrome (CS) in bilateral thighs, legs, forearms, and hands. This report is followed by a review of the literature regarding the pathophysiology of this rare sequela of cardiogenic shock. While the progression from cardiogenic shock to AbCS and ultimately to PCS has been hypothesized, no prior case reports demonstrate this. Furthermore, this case suggests more generally that IAH may be a risk factor for extremity CS. Future studies should examine the potential interplay between IAH and extremity CS in patients at risk, such as polytrauma patients with tibial fractures.

## Introduction

First described in 2007, polycompartment syndrome (PCS) is the combination of symptoms resulting from pathologically increased pressures in multiple regions of the body, including the thorax, abdomen, extremities, head, pelvis, and associated sub-compartments [1]. Increased pressures result from a range of etiologies, including traumatic and atraumatic, and lead to widespread organ damage from decreased perfusion of bodily tissues [2]. In cardiogenic shock etiology, rising compartmental pressures result from venous congestion pushing fluid into the interstitium, a phenomenon that can be exacerbated by massive fluid resuscitation. Such a condition can be quickly fatal as a self-perpetuating cycle of increased pressure sets in, contributing to organ damage and further fluid retention in compartments throughout the body. Simultaneously, hypoperfusion stemming from cardiogenic shock worsens organ damage. However, the paucity of cases in the literature complicates efforts to better diagnose and treat PCS.

We discuss a rare case of a patient who developed PCS secondary to cardiogenic shock. In this patient, it is hypothesized that venous congestion and fluid resuscitation led to intra-abdominal hypertension (IAH) with the simultaneous development of compartment syndrome in the abdomen and multiple extremities. There have been no previous reports of cardiogenic shock-related compartment syndrome in the extremities. Excluding the extremities, cases of cardiogenic shock-induced abdominal compartment syndrome (AbCS) alone are exceedingly rare, with only a handful of cases in the literature [3,4]. This case highlights cardiogenic shock as an etiology of secondary AbCS and suggests a possible interaction between IAH and extremity compartment syndrome.

## Case presentation

A 53-year-old male with a past medical history significant for nonischemic dilated cardiomyopathy with biventricular dysfunction, symptomatic premature ventricular contractions (PVCs), hypertension, hyperlipidemia, and obesity presented to an outside institution first with a ventricular fibrillation (VF) cardiac arrest. His initial cardiac arrest occurred near his home, where he was found down for approximately eight minutes before a bystander nurse began cardiopulmonary resuscitation (CPR). When emergency medical services arrived, they found him to be in VF and performed CPR with the return of spontaneous circulation (ROSC). Upon arrival at a community hospital, he suffered five additional VF cardiac arrests, each lasting around 10 minutes, before achieving ROSC. He required intubation, sedation with propofol, and high-dose pressors for blood pressure maintenance, including arginine vasopressin (0.04 mcg/kg/min), epinephrine (0.08 mcg/kg/min), and norepinephrine (0.45 mcg/kg/min). His course was complicated by an aspiration event resulting in severe acute respiratory distress syndrome (ARDS). An intra-aortic balloon pump was placed for cardiogenic shock, and he was started on amiodarone, which was switched to a lidocaine drip due to persistent arrhythmias.

Three days after the initial cardiac arrest, he was transferred to our institution for consideration of venoarterial (VA) extracorporeal membrane oxygenation (ECMO) cannulation due to worsening cardiogenic shock. Evidence of cardiogenic shock included signs of increased preload (pulmonary edema, hepatomegaly), a reduced ejection fraction of 30% to 35%, known dilated cardiomyopathy, status post-cardiac arrest, and poor perfusion of organs and extremities. Immediately upon arrival to the cardiovascular surgical intensive care unit, his labs and vitals (pH 7.09; partial pressure of carbon dioxide (PaCO2) 95 mmHg; partial pressure of oxygen (PaO2) 57 mmHg; saturation of peripheral oxygen (SpO2) 88% on 100% fraction of inspired oxygen (FiO2); lactate 4.6 mmol/L) showed signs of a severe hypercarbic respiratory acidosis with a lactic acidosis, as well as signs of acute renal failure (ARF) (serum creatinine 5.0 mg/dL, baseline 1.2; K; 5.8 mmol/L). The patient was placed on dialysis. These lab trends are displayed in Table 1 below.

**Table 1:** Patient labs tracking mixed respiratory and metabolic acidosis and ARF Daily pH, PaCO2, and PaO2 values are given as per the lowest measured pH value of the day. PaCO2: Partial pressure of carbon dioxide, PaO2: Partial pressure of oxygen, SpO2: Saturation of peripheral oxygen, ARF: Acute renal failure, K: Potassium

Day of hospitalization	pH arterial	PaCO_2 _arterial (mmHg)	PaO_2 _arterial (mmHg)	SpO_2_ (%)	Serum creatinine (mg/dL)	K^+^(mmol/L)	Urea nitrogen (mg/dL)
Day 1	7.09	95	57	88	5.0	5.4	39
Day 2	7.03	95	73	74	3.4	5.6	27
Day 3	7.09	74	78	96	2.3	5.2	22
Day 4	7.13	69	70	93	2.0	4.6	24
Day 5	7.27	56	102	94	1.8	4.0	26
Day 6	7.28	53	64	100	1.6	4.2	28
Day 7	7.32	46	69	100	1.7	4.5	33
Day 8	7.24	60	70	100	1.4	4.8	31
Day 9	7.20	68	103	98	1.7	-	47
Day 10	7.26	53	72	100	1.7	4.8	46
Day 11	7.30	53	67	99	1.4	4.7	37
Day 12	7.33	49	91	100	1.4	4.4	29
Day 13	7.25	58	85	98	1.5	5.0	29
Day 14	7.15	73	93	100	1.6	4.1	39
Day 15	7.29	53	79	100	1.2	4.2	30
Day 16	7.39	40	99	100	1.3	4.3	30

Given his anuric ARF, difficulty with ventilation despite elevated airway pressures, and tense, distended abdomen on physical exam, there was a concern for AbCS exacerbated by aggressive resuscitation, which included 10 liters IV in the 24 hours after transfer to our institution, for what appeared to be septic shock complicating his cardiogenic shock. Table 2 outlines the patient's daily intake and output during hospitalization at our institution. Clinical signs of AbCS included elevated bladder pressure (26 mmHg), increasing airway pressures on ventilation (plateau 38 mmHg; peak airway pressure 75 mmHg), and anuria. Ultimately, he was deemed not a candidate for ECMO due to his severe thrombocytopenia (platelet count 13 K/mm3), acute liver failure (aspartate transaminase 156 U/L; alanine transaminase 57 U/L; albumin 2.1 g/dL; total bilirubin 2.6 mg/dL), and ARF.

**Table 2:** Patient fluid intake and output broken down by modality All units listed are in milliliters (mL)

Day of hospitalization	Intake							Output					Net input/output
	Oral	Intravenous (IV)	Nasogastric (NG) tube	IV piggyback	Total parenteral nutrition	Tube feeding	Total intake	Urine	Emesis/NG output	Ultrafiltration	Negative pressure wound therapy	Total output	
Day 1	0	1004	0	0	0	0	1004	50	0	0	0	50	954
Day 2	0	12585	0	850	0	0	13435	15	0	103	2200	2318	111177
Day 3	0	8716	0	1450	0	0	10166	0	0	20	6400	6420	3746
Day 4	0	3978	100	800	0	0	4878	0	0	586	6875	7461	-2583
Day 5	0	2547	30	350	0	0	2927	0	0	1260	7600	8860	-5933
Day 6	0	3310	60	900	0	0	4270	0	100	1618	8050	9768	-5498
Day 7	0	1644	180	1300	0	0	3124	0	150	776	6350	7276	-4152
Day 8	0	2070	210	1050	230	0	3560	0	0	1117	4875	5992	-2432
Day 9	0	265	30	250	414	0	959	0	100	239	850	1189	-230
Day 10	0	396	0	300	621	0	1317	0	400	1849	450	2699	-1382
Day 11	0	298	0	250	345	0	893	0	0	525	0	525	368
Day 12	60	611	0	300	0	120	1091	0	0	1287	0	1287	-196
Day 13	0	1231	0	1050	187	0	2468	0	0	1147	900	204	421
Day 14	0	1464	0	800	1360	0	3624	0	250	3490	1175	4915	-1291
Day 15	280	435	90	1700	1632	115	4252	0	100	6464	550	7114	-2862
Day 16	0	258	120	1000	952	40	2370	0	900	2374	100	3374	-1004

The day after transferring to our institution, the patient was deemed to have AbCS and was emergently brought to the operating room as a level 1 case for decompressive laparotomy. Intraoperatively, the general surgery team noted a relatively normal-appearing bowel and a liver that had looked pale at the beginning of the procedure but regained color upon opening of the abdominal compartment. The surgery team was concerned that he had developed bilateral lower and upper extremity compartment syndrome (CS) as well based on the tense appearance of his limbs; orthopaedic surgeons were consulted to assess Stryker pressures. They subsequently performed bilateral thigh, leg, forearm, and hand fasciotomies. Immediately after abdominal decompression, his peak airway pressure declined from 75 mmHg to 50 mmHg, suggesting a decline in plateau pressures from changes in abdominal pressures after laparotomy. Furthermore, the liver appeared pale and ischemic, which, in conjunction with the elevated liver enzymes, suggested a shocked liver. 

**Figure 1:**
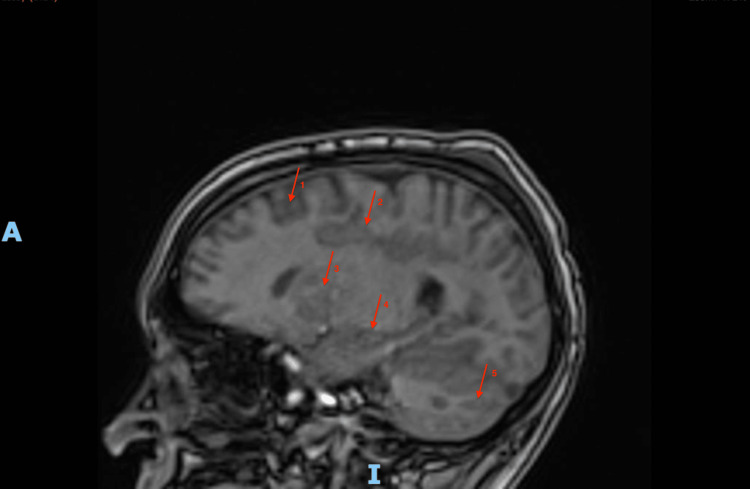
Brain magnetic resonance imaging shows areas of restricted diffusion scattered throughout the cortex (arrow 1), subcortical white matter (arrow 2), periventricular white matter (arrow 3), deep gray matter (arrow 4), and cerebellum (arrow 5). These likely represent a combination of watershed and ischemic infarction

On postoperative day 1, his acidosis improved mildly (pH 7.16; PaCO2 68 mmHg; PaO2 68 mmHg). His ARDS (pCO2 25 mmHg; blood pH 7.29), hypoperfusion, and ARF (creatinine 1.5 mg/dL; potassium 5.0 mmol/L) also improved in the following days, but his clinical picture remained highly unstable. As displayed in Figure 1, brain imaging revealed significant hypoxic-ischemic brain injury, and auditory and EEG activity were non-reactive to external stimuli, portending a grim prognosis. His abdominal fascia was closed six days after decompressive laparotomy, and his extremity fascia and wounds were closed 11 days after fasciotomies. A recovery remained unlikely, and he ultimately passed away 16 days after his initial hospitalization from an episode of VF without ROSC.

## Discussion

**Figure 2:**
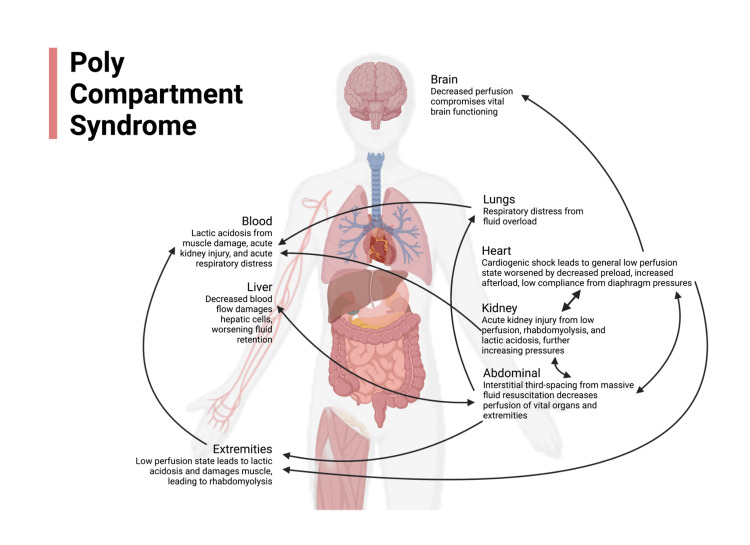
An illustration of the combination of cardiogenic shock and massive fluid resuscitation in our patient that kicked off a vicious cycle of increasing pressures and acidosis affecting vital organ function throughout the body. Figure created by authors using BioRender (Science Suite Inc., Toronto, ON, CA)

Patients in cardiogenic shock often exhibit IAH due to rising venous pressures leading to fluid interstitial third-spacing [5]. This multi-factorial etiology of IAH is believed to be exacerbated by rapid delivery of fluid boluses and administration of high-dose vasopressors to maintain arterial pressures [2], all worsened in this case by the presence of a distributive shock component and a high degree of inflammation (lactate dehydrogenase 1619 U/L). Subsequently, IAH is recognized in the theoretical literature as a precursor to and central driver of compartment syndrome in the extremities [6]. As outlined in Figure 2, IAH can compromise vital organ function, which further diminishes perfusion to the extremities. In the kidneys and liver, this happens as IAH decreases blood flow, contributing to the development of acute kidney injury (AKI), declining hepatic cell function, and fluid retention [6-8]. In this case, the patient’s markedly elevated creatinine and liver function enzymes demonstrate the rapid onset of multi-system organ failure in association with severe IAH. Intra-abdominal hypertension can also decrease cardiac compliance due to the upward displacement of the diaphragm, which compresses the heart [6]. Furthermore, IAH is hypothesized to increase afterload and decrease preload, resulting in low cardiac output, tachycardia, and worsening perfusion of the extremities [6,9].

The vital organ dysfunction from IAH can be classified as AbCS if intra-abdominal pressures are sustained above 20 mmHg [2]. Abdominal compartment syndrome may serve as a checkpoint in the progression to PCS involving the extremities. Malbrain et al. explain how AbCS can decrease perfusion to muscle tissue, eventually causing ischemia and contributing to fluid interstitial third-spacing [6]. This is hypothesized to be a self-perpetuating cycle: as intrabdominal pressures continue to rise, the central venous system is compressed, resulting in high venous pressures, which further perpetuates fluid retention by reducing renal blood flow and worsening elevated compartment pressure in the extremities [6,10]. Additional factors such as the administration of high-dose vasopressors, massive fluid overload, a mixed distributive shock picture, and inflammation can all contribute to declining extremity perfusion. Abdominal compartment syndrome can also result in respiratory distress, which exacerbates hypoxemia and brings on a mixed metabolic and respiratory acidosis, potentially further damaging muscle tissue. Much of the literature’s theoretical description of PCS pathophysiology mirrors the patient’s clinical course in this case [6]. Furthermore, the patient in this case also exhibited risk factors for IAH and AbCS, which include obesity, shock, placement on mechanical ventilation, and positive end expiratory pressure >10 mmHg [11-14].

Due to the central role that IAH and AbCS are hypothesized to play in PCS, understanding the diagnosis and treatment of AbCS can help mitigate the risk of compartment syndrome progression to the extremities [6]. Like compartment syndrome arising elsewhere in the body, AbCS can be classified as primary, resulting from abdominal trauma or abdominal surgical complications, or secondary, resulting from a condition originating elsewhere in the body causing transmission of high pressures into the abdomen [6,9]. However, current guidelines for treatment of AbCS, not necessarily involving other compartments, focus on the primary etiologies of the syndrome [9,11,15]. Naturally, case reports of AbCS also largely focus on primary AbCS instead of the rarer cardiogenic shock cause presented herein [16-19]. Even among the secondary AbCS literature, studies typically investigate cases with trauma and/or burns outside of the abdomen [13,20-25]. While there are similarities, risk factors, injury and illness patterns, treatment protocols, and pre-ICU courses can vary widely among the various etiologies of AbCS [20]. In addition, cardiogenic shock AbCS is likely much more frequent than commonly believed due to decreased awareness of the atraumatic etiologies of compartment syndrome [26]. Thus, further case reporting of cardiogenic shock AbCS is of value to better diagnose, prevent, and treat patients before further progression to PCS involving the extremities.

Both PCS and secondary AbCS are typically associated with a critical illness requiring massive fluid resuscitation and are widely recognized as highly lethal [2,3,26,27]. Over-resuscitation works in tandem with cardiogenic shock to cause edema and intra-abdominal fluid accumulation, raise intra-abdominal pressure (IAP) and extremity pressures, and lead to a general low perfusion state, which activates the renin-angiotensin-aldosterone system, further overloading the body with fluids [28]. In particular, crystalloid fluids such as dextrose solutions and lactated ringers have been linked to exacerbated respiratory and cardiac complications due to significant disruption of cell volume, which alters regulatory mechanisms of the inflammatory cascade [20,27]. In our patient, the exact fluid resuscitation volumes delivered before the decompressive laparostomy are unknown, as these were given before transfer to the authors’ institution. Nevertheless, over-aggressive delivery of fluids must be monitored in cases of IAH to prevent AbCS and, eventually, PCS. This is particularly true in cases where additional risk factors are present, including the administration of high-dose vasopressors, distributive shock, and a high degree of inflammation.

The definitive treatment for the abdominal component of PCS is decompressive laparostomy with gradual fascial closure, while fasciotomy is the standard of care for extremity CS [29,30]. In cases of cardiogenic shock PCS, early initiation of these surgeries or equivalent therapy before the self-perpetuating cycle of multi-system organ failure sets in is emphasized to prevent syndrome progression [4]. Our patient underwent laparostomy three days after cardiac arrest, two full days later than in a similar case in which AbCS did not progress to PCS and which resulted in patient survival [4]. There exist successful cases of alternative treatments for secondary AbCS, but the underlying theme is rapid recognition of the syndrome within 24 hours and initiation of therapy [4,31].

## Conclusions

This case highlights the risk factors and treatment outcomes of cardiogenic shock-related compartment syndrome that can spread to the extremities. Greater awareness can help clinicians make informed decisions about the quantity of fluid resuscitation and a quicker diagnosis and treatment of this syndrome. A direct study of the impact of IAH on the development of extremity compartment syndrome is warranted. Because of the link between IAH and progression to PCS in this cardiogenic shock patient, it is hypothesized that similar pathophysiology makes IAH a potential risk factor for the development of extremity compartment syndrome in general at-risk populations, such as polytrauma patients with tibial fractures. In addition, there is much more to be elucidated through further clinical case studies of cardiogenic shock, AbCS, and the pathway toward PCS involving the extremities.
